# Exploring staff conceptions of prevention and management practices in encounters with staff-directed aggression in supported housing following education and training

**DOI:** 10.1186/s13033-020-00387-2

**Published:** 2020-08-08

**Authors:** Erlend R. Maagerø-Bangstad, Knut Tore Sælør, Ole Greger Lillevik, Ottar Ness

**Affiliations:** 1 Dept. of Health and Social and Welfare Studies, Faculty of Health and Social Sciences, University of South-Eastern Norway, Centre for Mental Health and Substance Abuse, P.O. box 7053, 3007 Drammen, Norway; 2 Dept. of Health and Care Services, Faculty of Health Sciences, UiT The Arctic University of Norway, P.O. box 385, 8515 Narvik, Norway; 3 Dept. of Education and Lifelong Learning, Norwegian University of Science and Technology, P.O. box 8900, 7491 Trondheim, Norway

**Keywords:** Staff-directed aggression, Prevention, Management, Practice, Mental health, Supported housing, Education, Phenomenography

## Abstract

**Background:**

Staff-directed aggression is a concern for service providers in mental healthcare, frequently affecting both the quality of services and staff wellbeing. This also applies to supported housing services for people with mental health problems. Staff themselves consider training to be an important route to improve the prevention and management of staff-directed aggression. The aims of this study are to explore how staff in community mental health supported housing services conceptualize practice in prevention and management of aggression and how these conceptions develop following a local education and training endeavor in disempowerment-sensitive, de-escalating and knowledge-based risk assessment and management.

**Method:**

Phenomenography, a qualitative research approach, was adopted to pursue the study aims. The data consisted of 26 semi-structured interviews with 13 participants from five different municipal housing facilities in Oslo, Norway. Participants were interviewed on two occasions, once prior to participation and once subsequent to the finalization of the education and training sessions.

**Results:**

The analysis led to the development of six qualitatively different, yet logically interrelated, categories of description regarding practice in encounters with staff-directed aggression: (1) *Observation, alertness and awareness*, (2) *Established understanding and knowledge of service users*, (3) *Team*-*based risk management and deliberation*, (4) *Adaption of own dispositions and behaviors*, (5) *Reflexivity, sensitivity and care* and (6) *Involvement and dialogue*. These conceptions were found to vary in meaning and focus; they ranged from implementing safeguarding and protective measures, to drawing on what was portrayed in terms of staff’s expert knowledge, to increasingly allowing for, and committing to, tenant perspectives in designing practice. The results indicate a moderate, yet beneficial, effect of the course on conceptual change in the participants.

**Conclusion:**

This study shows that practice in encounters with staff-directed aggression is conceptualized as complex and multifaceted by staff in mental health supported housing services and that the various conceptions have different implications for the way staff-directed aggression is mitigated individually and collectively. Our findings also suggest that it is beneficial to take conceptual variation regarding practice into consideration when devising education and training aimed at enhancing staff knowledge, skills and practices.

## Background

Internationally, the de-institutionalization trend in mental health has led to various supported housing schemes for people with mental health problems [1]. In Norway, under the national action program for mental health [2], people described as having severe mental health problems are predominantly offered accommodation in supported municipal housing, if unable to rent or own in the private market [3]. Such supported housing generally implies provision of some kind of community-based mental health or psychosocial service, ideally tailored to the tenant’s individual needs for social and practical support. The context of this study is supported housing services based on type 1 in the Simple Taxonomy for Supported Accommodation (STAX-SA) [4]: (a) *staff on*-*site*, (b) *high support*, (c) *limited emphasis on moving on*, and (d) a *congregate setting*. As this bears some resemblance to inpatient settings, community supported housing has been claimed to carry the risk of adopting ‘institution-like’ qualities [5], despite the initial intention to promote ‘normality’ and social inclusion of tenants. Dyb [5] further suggests that the organization of the services, and whether the staff mainly understand their work in terms of providing institutional or home-based care, affect whether tenants experience their living situation as resembling an institution or a home.

There is evidence to suggest that people with mental health problems are not violent [6, 7]. Rather, people experiencing mental health or substance abuse problems are often victims of violence [8, 9]. Nevertheless, mental health staff sometimes encounter violent behavior and aggression from service users. International studies have revealed high levels of staff-directed aggression in mental healthcare [10, 11]. In a recent Norwegian study, 51% of mental health staff and 55% of substance abuse staff had experienced violence in the previous 12 months [12]. However, Campbell [13] finds non-institutional workplace violence to be severely under-researched; there is therefore little knowledge of staff-directed aggression in primary mental healthcare or its impact on service provision. One noticeable exception is a recent review that among other showed that one-third of tenants in supported housing displayed aggression [14].

Studies show that staff-directed aggression negatively effects both the quality of care provided by mental health staff [15] and their interpersonal job performance, including affective commitment, increased negligence and impaired cognitive functioning [16]. Further, aggression tends to harm the staff-service user relationship [17], and decrease the duration and frequency of visits in home-based services [18]. Violence toward mental health staff often leads to involuntary hospital admissions [19] and subsequent coercive treatment [20].

### Study context

Staff consider training in the prevention and management of staff-directed aggression to be a key mediating factor in violent encounters with service users [21]. During 2018, Oslo Municipal Health Agency developed and implemented locally based training and education for staff in supported housing facilities in three municipal districts. The aim was to increase knowledge and skills in the prevention and management of staff-directed aggression utilizing non-physical de-escalation [22], disempowerment- and disequilibrium sensitive [23, 24], and recovery-oriented [25] principles. First, there was a common introductory course, followed by two local sessions at each facility. Designated resource persons from each facility collaborated in developing the courses and sessions based on their assessment of the local requirements at their workplace. The local sessions were designed to provide further elaboration of aspects of prevention and management of staff-directed aggression and violence presented during the introductory course and bestow participants with a venue for learning via inter-collegial practice-based reflection and scenario-based training. The sessions took place at different intervals in each housing facility, and the implementation period lasted from February 2018 until March 2019.

The aim of this study is to increase knowledge of how mental health workers in locally based education and training conceive prevention and management practice in aggressive or violent encounters with tenants, and how these conceptions develop during the competence-development activities. In addition to enhancing understanding of the particular education and training activities, we have aimed to contribute to the current knowledge base, or ‘collective mind’ [26], regarding experiences and conceptions of non-institutional, staff-directed aggression. The specific research questions we have developed are:

1. How do staff in mental health supported housing attending locally based education and training in prevention and management of staff-directed aggression conceptualize prevention and management practices in encounters with aggressive clients?
2. How do the participants’ conceptions of practice develop following education and training in prevention and management of staff-directed aggression?

By virtue of persons living in supported housing facilities being both tenants and users of community mental health services the terms ‘tenant’ and ‘service user’ will be used interchangeably throughout this article.

## Methods

To explore the participants’ conceptions of the phenomenon of practice in the prevention and management of staff-directed aggression, we utilized research tools from phenomenography. We have been inspired by Micari, Light, Calkins and Streitwieser [27] in our exploration of developments in how the participants conceived practice, in order to assess the impact of the training and education.

Marton and Booth [28] state: “Phenomenography aims to reveal the qualitatively different ways of experiencing various phenomena” [p. 136]. Thus, the outcome of a phenomenographic analysis is typically an ‘outcome space’ consisting of descriptive categories, or ‘ways of seeing’ regarding a phenomenon, i.e. “the complex of categories of description comprising distinct groupings of aspects of the phenomenon and the relationships among them” [28, p. 125]. These categories describe the experiential and conceptual characteristics of a certain phenomenon according to the way conceptions are given meaning for persons, the ‘referential aspect’, and how conceptions are structured and how this relates to the other components in the outcome space. A phenomenographic outcome space is often hierarchical and comprises logically inclusive and interrelated categories of description.

The underlying rationale behind the hierarchical presentation in phenomenography is that conceptions develop from less advanced ways of seeing to increasingly more compound and comprehensive considerations of a phenomenon. Further, Marton and Booth [28] contend that more complex comprehensions of a phenomenon enable an individual to hold multiple important aspects simultaneously in awareness when encountering a phenomenon, and thus implying a capacity for more powerful ways to handle both familiar and novel situations.

Lum [29] describes an expansive mode of assessment where a wide array of evidence is considered in evaluating people’s competence, and ‘judgements of significance’ are made in ascribing value to the evidence. Such assessment is suitable for qualitative methodologies and we support this notion in the rationale underlying this study.

Causal dispositionalism [30] is a recent ontological approach to causality granting qualitative and complex data primacy and seeking to establish mechanistic knowledge in establishing causation. Since phenomenography is a qualitative research approach geared toward complexity, we find it as such, appropriate for discerning facets of impact from educational interventions in this study.

### Design

The study was designed within a descriptive-explorative framework. Phenomenography has been described as a data-driven and empirically oriented research approach [31]. However, we view research, scientific reasoning, and the specific research approach, in line with Sandberg and Taragama [32], in terms of a social constructionist framework where knowledge is co-constructed in a communal effort involving researchers and study participants [33]. On an axis between concrete or abstract analysis of either purely manifest or latent content [34], we would place this study as being moderately interpretative and concrete. We have aimed at providing an as representative account of the participants’ ways of seeing in the developed categorizations. Nevertheless, we also concede that the participants themselves might have difficulty in recognizing their own understandings of themselves or their work from our descriptions.

A consultative reference group was established to inform various stages of the research process. It consisted of former service users of community mental health and substance abuse services, managers and staff from mental healthcare and a representative from a collaborating specialist psychiatric service. Most notably, they helped develop the interview guide and contributed to the final stages of the analysis. The reference group had what Borg [35] terms an advisory position, neither determining aims nor research methods but nevertheless contributed valuable insights in the research process and furnished our findings with transferability and relevance to practice.

### Recruitment and participants

Since phenomenography aims at variation between people in ways of seeing, sampling strategies suitable for obtaining maximal variation are preferable; we accordingly chose criterion-based, purposeful sampling [36], seeking to maximize experiential and conceptual variation between participants.

After the first author had approached managers or resource persons from mental health and substance abuse services with written information about the study and a request to recruit participants, 13 staff members (ten females and three males) from five mental health supported housing facilities in two districts agreed to participate in the study. The service providers were approached by e-mail and received written information about the study. Variation was sought in the full-time equivalent status of the participants, their relevant work experience, position, level of formal education, and gender. The participants ranged from 28 to 61 years (median 44 years) of age. Their relevant work experience in mental health varied from less than 1 year to over 20 years. The level of formal education ranged from no relevant education to completed education in general nursing, social work or social education. Eight participants had experienced workplace violence or threats directed at them and seven had witnessed violence or threats directed at colleagues. Three participants had either witnessed or been subjected to violence or threats outside of work. Only one participant reported no experience of violence or threats of violence, while one participant either did not know or opted not to answer.

### Data collection

Semi-structured interviewing is considered the preferred phenomenographic data collection strategy [37], and the interview questions were open and inquisitive, based on an interview guide developed in collaboration with the reference group. All participants were interviewed once before the introductory course and once two–18 weeks after the final session in each housing facility, totaling 26 interviews. Most interviews were held within 8 weeks after the final sessions. The interview guides for the first and second interviews contained similar questions, but the second interviews also included questions regarding perceived change following the education. Herein, participants’ own experiences were revealed, and sometimes, their thoughts regarding their own competence, the workplace atmosphere and their relations to colleagues and managers. All interviews were conducted at the participants’ workplaces, except for one phone interview, for practical reasons.

The first interviews lasted from 50 to 90 min (average 1 h), while the second ones took 70–120 min (average 90 min). All interviews were recorded and transcribed verbatim.

### Data analysis

Traditional phenomenographic analysis is decontextualized [28, 38]. In phenomenography, all interview statements about a particular phenomenon are considered as comprising an analytically purposeful ‘pool of meaning’ [28] regarding that phenomenon, and the ensuing analysis consists of the researcher(s) grouping and categorizing apparently related statements in the data. Thus, all meaning units from each interview were included in the ‘pool of meaning’ and treated equally regardless of whether they were found in the first or the second interview.

We adhered to the stepwise outline of a phenomenographic analysis provided by Sjöström and Dahlgren [39]. This included *familiarization* with the data, *compiling* answers to questions, *condensation*, *grouping*, preliminary *comparison*, *naming* of the categories and lastly, a *contrastive comparison* of the established categories.

The authors pursued clarification of their own preconceptions through repeated discussions and reflections about practice in encounters with staff-directed aggression, which is an example of dialogic reliability checking [37]. Additionally, the first author was committed to regular self-disclosure and critical evaluation of his own attitudes and preconceptions regarding staff-directed aggression and prevention and management practice throughout the entire study. Pragmatic and communicative validity was sought through internal discussions between the authors on the content and coverage of the categories established, through consultations with the reference group during the final stages of analysis and finally, in a seminar for community mental health staff, managers and service users, where preliminary categorizations were presented and discussed. Such procedures are examples of member checking [40], which we used to enhance trustworthiness in the study findings. Categorization was completed with the construction of six categories of description regarding the participants’ understanding of practice in encounters with staff-directed aggression and violence.

Unlike traditional phenomenographic analysis, where “individual voices are not heard” [28 p. 114] and descriptions of variation in a population are limited to the collective level, the first author finally reviewed the data to identify conceptual changes in the answers from individual participants. A threshold value of three individual statements regarding an aspect of a particular conception was chosen as sufficient to indicate that a participant had acquired a particular way of seeing the phenomenon. Statements from the first interview, and later from the second interview, that could be linked to any of the six categories were identified. A comparison of these showed how the participants’ focus had changed between interviews. Marton argues that learners’ ability to express a conception for the first time signals a development in their ability to see the phenomenon in a particular way [38]. The findings from the last part of the analysis might therefore give us a valuable indication of possible shifts in participants’ focus and awareness regarding practice in prevention and management of staff-directed aggression from the first interview to the second. This might also reveal any impact from participation in the education and training.

### Ethics

The Norwegian Centre for Research Data recommended this study (Case No. 542044). Before the first interview, written informed consent was obtained from all participants. Before both interviews, the interviewer provided a brief summary of the same information, stating that participation was voluntary and that withdrawal would have no negative repercussions.

Confidentiality was a topic of considerable importance in this study, particularly since all but one interview were conducted at the participants’ workplaces, and there were descriptions in all interviews of specific instances of staff-directed aggression involving specific service users and either the participants themselves or some of their colleagues. We removed any potentially identifying characteristics from the material when writing up the findings.

Another ethical consideration was the potential for re-traumatization of participants when recounting aggressive incidents with tenants. The interviewer therefore needed to be wary of signs of distress from the participants. Although no participants appeared distressed during the interviews, some gave the interviewer the impression of being highly preoccupied with earlier experiences of victimization. Therefore, the interviewer, in an open and non-directive manner, paid particular attention to those participants and their urge to share in the remainder of their interviews. Qualitative interviewing can have therapeutic value [41]. Although this was not intended, the opportunity to talk about experiences to a stranger might have provided a welcome venting of previously unprocessed emotions from aggressive encounters with service users.

## Results

The analysis resulted in six qualitatively different, yet logically interrelated, hierarchical categories of description. Comparing the interviews, we found a modest conceptual development among the participants. The categories of description varied particularly with regard to what emphasis was placed on the different parties in the helping relationship and whose agency was favored in the prevention and management of staff-directed aggression. In this section, we confer our results. The categories are presented in Table 1. Each category is represented by the use of illustrative quotations, intended to convey important experiential dimensions of each conception, or particular way of seeing.

**Table 1:** Outcome space of participants’ conceptions of practice in staff prevention and management of staff-directed aggression and violence

Descriptive categories	Referential aspect	Structural aspect
1. Observation, alertness and awareness	Safeguarding under unpredictable and threatening circumstances, limited resources and staff disempowerment	Practice as protection
2. Established understanding and knowledge of service users	Adaptation to and restriction of tenant’s propensities for violence and aggression	Staff as knowledgeable and expedient authorities
3. Team-based risk management and deliberation	Developing solutions and strategies for management of risk in the workplace collective	Staff’s aggregate experience and knowledge as a basis for practice
4. Adaption of own dispositions and behaviors	Self-awareness and self-regulation are required in addressing situations involving staff-directed aggression	Using oneself to build non-violent relationships and interacting responsively with tenants
5. Reflexivity, sensitivity and care	Meeting aggression with self-critical and empathic consideration and respect towards the other	Practice attentive of tenant’s needs in the situation, experiences of disempowerment and providing reflexive care
6. Involvement and dialogue	Involving tenants in increasing understanding of aggression and in developing preventive and management measures	Tenants and staff as equal partners in the helping relationship

### Observation, alertness and awareness

Most participants described practice in encounters with staff-directed aggression in terms of ‘observation, alertness and awareness’. Their workplace was conceived as involving considerable risk with highly unpredictable, unique situations involving aggression from primarily psychotic and unstable tenants. Considerable energy was invested by staff in keeping themselves and their colleagues alert and vigilant in observing the movements of the tenants. Attention by staff was revealed as highly situationally dependent, vulnerable and fickle, and was reported to decrease in the wake of aggressive encounters. Some stated that this was due to the staff’s need to rest and regroup following alarming incidents. Routine practices also appeared to make staff become negligent and unresponsive to observable signs of aggression in clients. Especially when staff were uncertain of the risk of aggression from tenants, the need for being alert and attentive was described as particularly important:

*“It wasn’t a pleasant atmosphere in the facility. Not at all. When you went to work, and you were working, your shoulders never dropped. You had your guard up, all the time. We* [the staff] *agreed that ‘we don’t drop our shoulders until we’re done for the day’. Because suddenly* [snaps his fingers] *something happens, out of the blue.”* (M3, second interview).

Becoming habituated to staff-directed aggression, through regular exposure at work, was yet another threat to beneficial prevention and management practices that participants cautioned. The antidote to inattentive habituation was vesting mental energy in promoting awareness.

Participants mentioned preparation and rehearsal as helping to manage aggressive encounters with tenants when their observation and “reading” of tenants’ behaviors seemed to have failed. Several participants stated that the locally based education and training activities had helped to maintain their focus on staff-directed aggression at work.

Participants often expressed disempowerment regarding staff-directed aggression, due to lack of influence on the composition of tenants in the facility, inadequate tools to address resistance and challenges presented by uncooperative tenants, and poor job alternatives for staff intending to leave. Service user autonomy was seen as potentially impairing staff interaction with tenants perceived in need of help to prevent deterioration and subsequent increased risk of violence. A perceived reluctance to interact with staff is seen in a rather typical statement from one participant regarding an ‘uncooperative’ tenant:

*“I don’t know if he actually has the necessary insight into his own limitations to understand that if he’d been more receptive towards receiving assistance or accepted guidance and counseling from us, he might have become more self*-*reliant. Because he really wants to manage most things by himself.”* (F10, first interview).

Exponents of this view held confidence in external interventions to solve situations of staff-directed aggression. This entailed involving managers to provide authority, treatment and sometimes including physical restraint, or the police in grave situations. Ultimately, when all possibilities to establish a helping relationship seemed exhausted, or a serious violent incident had transpired, the only available solution in this view was eviction, or forcing the tenant to move to other accommodations. This was something several of the participants endorsed.

### Established understanding and knowledge of service users

In conceptualizing prevention and management practice in line with the descriptive category of ‘Established understanding and knowledge of service users’, participants’ focus shifted toward the staff’s professional and experiential knowledge of tenants, and the staff as the primary originator of preventive and managerial strategies. Knowledge of individual triggering and response patterns was typically established over time, primarily by staff spending time with tenants and witnessing their behavior in various contexts. The staff’s assessments of risk were from this conception based on more or less formal diagnostic criteria and hearsay from colleagues or others. When familiar with a service user’s identifiable signs of aggression, the staff could better implement interventions and calm aggressive tenants.

*“I know this tenant a bit already. I think that’s a factor, because then you can see when he’s grumpy and avoid placing the two in the same room, you see? Try to avoid it, but you can’t be everywhere, can you?”* (F1, first interview).

In this view, the root causes of staff-directed aggression are found primarily in service user deficiencies, such as poor coping resources, communication or cognitive difficulties and psychotic traits. Staff-directed aggression is seen as a form of communication, related to service users’ frustrations reaching a threshold. Aggressive behavior is usually considered as rooted in tenants’ traumatic childhood experiences. Medication is the preferred intervention and violence is often perceived as associated with medication non-compliance. This approach advocates staff control and the establishment of safe boundaries between staff and tenants.

In this view, limit setting is a valued and frequently mentioned intervention, albeit fraught with risk, as some tenants strongly oppose it. Accordingly, this necessitates particular caution when approaching such tenants.

Some participants reported that the competence-development activities and ensuing reflections on practice had signaled an implementation of redefined and more controlling practices toward particular service users during home visits.

Perceived as being more proficient in communication, staff were perceived as obliged to provide simpler communication settings for tenants and match their own communication with tenants’ level of activation and the particular situation. Tenants were considered highly dependent on staff, both in social interaction and in avoiding escalation of situations. When asked about the place for service user involvement in establishing measures to prevent staff-directed aggression, the participants envisioned mainly a conditioned form of involvement, usually based on tenants’ compliance with regulations, realization of their ‘wrongdoings’, and their perceived cognitive ability to acquire such insights, thus positioning staff as ‘gatekeepers’ of service user involvement.

Finally, from this conception participants valued distancing and detachment from service users to avoid being manipulated or harmed by their aggressive behavior; they described emotional and interpersonal closeness as increasing the risk involved in service provision.

### Team-based risk management and deliberation

In the category of ‘Team-based risk management and deliberation’, practice is described according to dimensions of the workplace collective. A common agreement on practice and assessment of tenant behavior was idealized in this conception. However, this agreement was often described as lacking and divisive practice was repeatedly depicted as the untoward norm at the workplace.

*“We’re different, we do our work differently, and some of us disapprove of this and that, and then the manager does something that others disapprove of. And the outcome is a big mess in the team, which isn’t very helpful”*. (M3, second interview).

Several participants cautioned against splitting and manipulative behaviors from tenants posing an additional threat to the team’s integrity and collegial collaboration.

However, several participants found dissimilar viewpoints to be valuable in analyzing episodes from different angles and enhancing staff understanding of tenants, as well as in finding good strategies for prevention and management of aggression. Collegial sharing of experiential knowledge was appreciated in this view, and was also depicted as important in evaluating incidents at work, necessitating openness and tolerance between colleagues. By observing colleagues’ strategies or modelling their practice to colleagues, participants valued mutual learning as a route for improving safety at work.

In this conception, the team provided support and care in periods of tenant aggression or following aggressive encounters. Several participants mentioned that violent incidents often make teams focus excessively on the perpetrating tenant, leading to fatigue in the staff and neglect of other tenants. Several also stated that the education and training helped the team maintain focus, even in more stable and uneventful periods.

Lone work was reported to involve greater risk, and participants regularly advocated working with colleagues when faced with challenging service users. This required mutual knowledge and understanding of the particular situations, as well as having congruous insight and skills in preventive and management practices in general.

### Adaption of own dispositions and behaviors

When participants conceptualized practice in prevention and management of staff-directed aggression in terms of ‘adaption of own dispositions and behaviors’, they did so with a clear recognition of the impact staff themselves can have on the development and manifestation of aggression. Participants spoke of the need to adapt their practice, behavior and bodily awareness according to tenant needs in aggressive encounters.

Strategies for preventing and managing service user aggression often mentioned in this approach are ‘containing’ tenants and being attentive of service user communication. Tenants being listened to and ‘seen’ by staff is presumed to make staff-directed aggression perceived unwarranted and thus de-escalate situations.

Staff behavior was considered particularly important in ensuring a safe working environment less likely to provoke clients. Staff should not respond to aggression in a confrontational, agitated, reproachful or vociferous manner. Participants valued considerate strategies, being respectful, inviting and “being sort of determined, but not too determined. So they feel they’re somewhat in charge of the situation themselves, without you overly controlling them” (F1, second interview). In this view, predictable and amicable staff and the ability to build trust installs in tenants a sense of security and confidence that they will receive consistent and unconditional care.

The focus in this conception is on staff characteristics such as experience-based insight into one’s personal boundaries and tolerance limits and the ability to uphold these in interaction with tenants. Some participants found that the education and training enhanced their ability to lower the threshold for threatening behavior and initiate preventive and management strategies at an earlier stage. Embodied sensitivity (or ‘gut feeling’) and a sense of own safety in situations were decisive for discerning risk in this view. Providing service users with descriptions or assertions about the situation or behavior could increase insight and prevent unmediated aggression.

*“What I think characterizes staff that are good at prevention, is that they dare to say something about what they see. Their prevention is sort of, ‘Now I can see it’s like this. What do you think about that? Am I seeing this in the wrong way, or do you think I’ve gotten it wrong now?” Because often thinking aloud can calm the client. So trying to acquire some reflection with the tenant, then…”* (F7, second interview).

Finally, time is an important factor in this conception. Staff must be patient and tolerant toward service users to improve their relationship and minimize aggression.

### Reflexivity, sensitivity and care

In the fifth category, ‘Reflexivity, sensitivity and care’, the prevention and management, and the explanations, of staff-directed aggression were portrayed as highly complex. In this conception, there was greater awareness of tenants and the interconnectedness between service users and staff. Staff were seen as responsible for monitoring their own attitudes and emotional reactions to the service users and for maintaining conscious reflection on their practice.

*“You have to be reflective. If you don’t reflect, things can quickly go wrong. Because you have to become aware of the things that happen here. We can’t always understand situations, or why someone reacts like this or that in a certain situation. In here, we’re talking about very disturbed people. They can have psychotic outbursts or whatever. But reflecting on our own behavior… That’s something we ought to be good at”*. (F2, second interview).

Prevention and management strategies were portrayed as based on a sensitive and active adaptation of staff members’ own reactions, behavior and attitudes to meet service user needs. What staff seemed to dread in this view was unreflective practice; the ideal was a reflective practitioner. Some called for reflection to be included more systematically in work routines. By extension, the ability to learn adaptively from situations distinguished skilled staff in this view.

Using sensitivity and empathy with tenants’ situation, and showing respect for their choices and considering them accountable, were considered prerequisites for implementing low conflict practices.

With the acknowledgment of service users’ right to privacy at home and autonomy in making own choices, respect appeared as a key value in this view: “I think that to establish a good working alliance with another person—and this goes for every human being —we need to be treated with respect” (F2, first interview). Empathic attention to the other’s perspective enabled staff to be understanding and compassionate in their interaction with tenants. It also provided another avenue for gaining insight into how their practices affected tenants, perceived as fundamental to adapting staff practices.

Some participants described violent tenants as relationally damaged. By deliberately showing that they cared about them as valuable human beings, staff imagined they could alleviate such relational damage and non-violent, trusting staff-service user relations could ensue. Some participants who subscribed to this view advocated close relationships between staff and tenants as a way to prevent aggressive escalation.

In line with an acknowledgment of phenomenal complexity and unlike the previous conceptions, some participants holding the fifth conception asserted staff-directed aggression in more positive terms as non-compliance and resistance from service users. Aggression was interpreted as service users still having enough spite in them to fight back and reject limitations imposed on them by both their own illnesses and repressive features of mental health services.

### Involvement and dialogue

In the sixth and final category, ‘Involvement and dialogue’, participants viewed staff and tenants as coequals in developing strategies for preventing and managing staff-directed aggression. Staff was urged to explore incidents in collaboration with tenants, and to help them express their experiences with and opinions on practice, thus mitigating the power imbalance in the service relationship, in a non-directive and caring atmosphere. Tenants’ reasoning behind their aggression should be included to enhance practice, and staff should prompt tenants to identify suitable strategies to cope with aggression, either alone or in collaboration with staff. Being mindful of how bodily activation impacts the ability to reflect and communicate for both parties, participants maintained that such dialogue ought to take place once the tenant’s level of activation had sufficiently decreased and stabilized.

Opportunities for learning for both parties present themselves by involving service users and reflecting with them on the causes and consequences of the aggression according to this conception. Through this, staff could increase their understanding of the tenant’s tolerance limits, triggers and preferred staff interventions, while tenants, in the other hand, could gain insight into their symptoms of aggressive escalation and develop individual management strategies. Improved insight into the consequences of their behavior would according to participants, enable clients to take greater responsibility for their actions. It was also mentioned that tenants could learn from gaining greater understanding of the reasons for staff safety procedures, thus making staff reactions to aggression more predictable and perceived as less threatening.

Dialogue following an incident was also described by some participants as having therapeutic and restorative potential. “I usually say there are always three sides to a story. You have your own experience, then you have the other’s experience and then you have the truth” (F10, second interview). By reflecting on a situation and allowing for both sides’ accounts of it, staff and tenants could potentially achieve closure and move on together.

In this view, participants valued being perceived as genuine in their care for tenants by promoting interpersonal knowledge and a forthcoming atmosphere, thusly aiding prevention of staff-directed aggression.

*“You should kind of play on their side. Not that ‘play’ implies that you’re at all fake. But show that you care. That you really want the best for them. You don’t want them to suffer. ‘I’m here if you need anything’, kind of. ‘I understand that you’re hurting right now’. Because you get a different kind of knowledge and get to know them in another way. And I think the service users here see that. I think they see far more than we think they do. If you genuinely care, that’s very important for cooperation between you and the tenants*.*”* (F1, second interview).

Participants suggested that if staff was being perceived as humble, open about own failings and having the ability to ask forgiveness if they in some way had treated a client wrongly or misunderstood a client, then tenants would show a more tolerant and equitable attitude toward them. Participants conceived this as promoting trusting relations between tenants and staff, enabling staff to communicate hope, promote change and persevere in providing care and support to help tenants move on in their lives.

### Conceptual changes identified in the data

When asked about their perceptions of the impact from attending the education and training sessions in the second interview, several participants mentioned increased awareness and reflection on the topic of staff-directed aggression in the workplace, both personally and collectively. Others were more negative toward the notion of change, often because of ostensibly vast differences in opinions on appropriate practice, inattention and even age-related inflexibility toward staff-directed aggression among colleagues. However, as can be seen in Table 2, only four participants eventually made statements in the second interview salient enough to be considered as advanced conceptions. The advancement in ways of seeing occurred as a one-step movement between the fifth category and the sixth. Interestingly enough, by the time of the second interview, two participants voiced previously unmentioned aspects of the phenomenon in line with the first conception. This implied they actually had experienced a downward movement in the hierarchical outcome space. One of these participants additionally experienced an upward development in her way of seeing, reaching the sixth conception by the second interview.

**Table 2:** Expressed conceptions identified prior to and following the education and training

Category	Pre-training	*N*=	Post-training	*N*=
1	F1, F2, F3, F4, F5, F6, F7, F10, M2, M3	*10*	F1, F2, F3, F4, F5, F6, F7, *F8, F9*, F10, M2, M3	*12*
2	F1, F2, F3, F4, F5, F6, F7, F8, F9, F10, M1, M2, M3	*13*	F1, F2, F3, F4, F5, F6, F7, F8, F9, F10, M1, M2, M3	*13*
3	F1, F2, F3, F4, F5, F6, F7, F8, F9, F10, M1, M2, M3	*13*	F1, F2, F3, F4, F5, F6, F7, F8, F9, F10, M1, M2, M3	*13*
4	F1, F2, F3, F4, F5, F6, F7, F8, F9, F10, M1, M2, M3	*13*	F1, F2, F3, F4, F5, F6, F7, F8, F9, F10, M1, M2, M3	*13*
5	F1, F2, F4, F5, F6, F7, F8, F10, M1	*9*	F1, F2, F4, F5, F6, F7, F8, F10, M1	*9*
6	F1, F2, F5, F6	*4*	F1, F2, *F4*, F5, F6, *F7*, *F8, F10*	*8*

Detected conceptual change between interviews: conception 1: 10  ⇒  12, conception 6: 4  ⇒ 8. Participants expressing previously unexpressed conceptions in the second interview are italicized

As previously mentioned, the first and second interviews differed substantially in both length and content.

## Discussion

This study has aimed to explore how mental health staff in supported housing conceptualize practice in prevention and management of staff-directed aggression. The six descriptive categories we identified in our data differ in complexity and in their structural and referential aspects. The categories are hierarchically disparate, yet logically inclusive, where each successive conception builds and expands on the preceding one.

Albeit from rather different contexts from the present study, one study [42] shows that staff training in prevention and management of staff-directed aggression and violence can have rather discouraging and even undesired effects. Much in a similar vein, Fry et al. [11] have argued that staff training and education is the typical answer to challenges posed by staff-directed aggression in mental health services, yet with rather dubious impact on practice. Notwithstanding, we will in the following discuss our findings in relation to other research on staff-directed aggression in mental health settings and potential implications for practice. We will also shed light on the conceptual changes identified in the study and factors that might have contributed to or impeded change.

### Differing explanations, differing practices

Duxbury [43] has showed how explanations of causes of staff-directed aggression are linked to staff behaviors toward service users. The underlying assumptions of reasons behind tenant aggression represented by the first three categories differ significantly from those of the other three in how they center on internal factors in the person. Focusing on internal factors, violence and aggression are chiefly explained with reference to perpetrator characteristics [44]. External explanations, however, refer to the environmental impact on aggression [45], ranging from the design of the housing, the regime and staff culture to the atmosphere in the housing. Interactional, or situational, explanations consider the impact of negative staff-tenant relationships in promoting aggression and violence [45]. The remaining three conceptions in our outcome space allow for a broader understanding, increasingly including external, interactional and situational factors in explaining aggression and providing suggestions for preventive measures. This view is supported by recent findings from research [46] and international recommendations [47]. As service user variables, such as psychopathology and substance abuse associated with aggression in mental health settings [45] are likely to be difficult to mediate, internal explanations have been shown to promote reactive and controlling management measures [43]. In contrast, more compound explanations pave the way for proactive strategies, which agree more with preventive practices [48]. In our study, it seems that the more compound conceptions of practice, including internal, external, interactional and situational variables, have provided staff with a broader range of strategies and approaches to tenant aggression than what is to be found through less compound conceptions.

### Staff-centered conceptions

The first category, ‘Observation, alertness and awareness’ was found to be intertwined with staff expressions of disempowerment when faced with aggression. Encountering tenants who seem unwilling to comply with regulations, treatment and other facets of service provision, staff might perceive themselves as unable to influence the situation or ensure their own safety. This conception involves reliance on external intervention and support by the healthcare organization to manage violence. Weingarten [49] has proposed that ill-advised practice, or even malpractice, can emanate when practitioners linger in an aware, yet disempowered witness position. This suggests that whenever expectations of external intervention are unfulfilled, staff will be more susceptible to intrusive and controlling practices toward tenants. Controlling and restricting behavior is often used by professionals in encounters with aggressive service users [50] and is frequently promoted in aggression management training programs [51], while such measures are also linked to paternalistic and coercive measures [20]. These are in themselves often perceived by service users as contributing to staff-directed aggression [51]. Bowie’s [52] typology of workplace violence includes aggression and neglect of service users as potential upshots of poor organizational resources and support. Shaw [53] points to financial priorities and the focus on efficiency in service provision as producing neglectful and even harmful staff behavior. Policies providing limited resources for care provision have also been identified as further hampering the development of staff-service user relationships and interpersonal knowledge [54], thus thwarting efforts to systematically prevent staff-directed aggression.

In the second descriptive category, ‘Established understanding and knowledge of service users’, disempowerment and perceptions of insecurity in work were mediated through the practitioners gathering information to gain an impression of tenants’ aggression potential. Risk is countered with limit setting, medication and communication devised to achieve tenant compliance with house rules. In this view, staff are the experts, knowledgeable in both establishing risk and devising appropriate measures to prevent violence and aggression. Duxbury and Whittington [51] find that many nursing staff endorse what they label traditional and biomedical management (i.e., medication and enforcing of rules). The expert position of the second conception is further developed into a collective level by the third conception, ‘Team-based risk management and deliberation’. In this understanding, the team is described as strongly influencing the development and maintenance of practice. As the originator of practice in this view, workplace culture and atmosphere will largely depend on how the supported housing services are conceptualized collectively. An understanding focused on practitioners will tend to downplay tenants’ views on appropriate practice in designing measures for prevention and management of aggression. Research on mental health service users’ perceptions of aggression shows that they link aggression to not being listened to or understood by staff [55]. Husum, Legernes and Pedersen [56] show how not being conceded participation or influence during mental health care makes service users feel humiliated, which further bolsters a sense of powerlessness associated with aggression [57, 58]. Feelings of being ignored and having one’s personal integrity violated by staff are argued to carry strong incentives for service users to respond aggressively as a self-defense mechanism in advocating their empowerment [55]. Shared problem solving is a central tenet in de-escalation [22], without which staff interventions are less likely to succeed in addressing aggressive escalation.

### Progressively tenant-centered conceptions

Related to this, in the fourth category, ‘Adaption of own dispositions and behaviors’, our participants seemed mindful of the interactional aspects of incidents involving staff-directed aggression and violence. Recognizing how their behavior in service provision might make tenants feel frustrated and disempowered, they acknowledged the need to adjust their own behavior to the requirements of the situation and de-escalation principles. Both Bowers [59] and Price and Baker [22] describe keeping calm as a prerequisite for de-escalation. Duperouzel [60] suggests that staff keeping calm, and thus not provoking aggression, will convey to service users that they can be trusted not to resort to violence in the situation, and promote self-esteem and positive emotions. Participants in a study by Carlsson, Dahlberg and Ekebergh [61] maintained that calm interpersonal communication by mental health staff, supported by corresponding body language, helps fostering non-aggressive relationships in mental health care. Participants voicing the fourth conception also found it important to devote time and attention to the tenant and the situation, which also seems to concur with service users’ view that the provision of time and space enables de-escalation [62].

A review of the literature on service users’ perceptions of aggression and management practices clearly calls for staff to be sensitive and responsive toward service users [55]. Practitioners have also been shown to endorse such a notion. Sensitivity is a key component in the description by Björkdahl et al. [50] of a particular aggression management style they call ‘ballet dancing’ and in acute mental health settings, sensitivity to patients’ individual triggers is central to recovery-oriented reduction of aggression [63]. In the present study, the conception “Reflexivity, sensitivity and care” also encompasses such insights. Lillevik and Øien [64] found that practitioners highlight wishing the best for the service user as a caring stance, communicating positive regard and promoting non-violence in the helping relationship. By accentuating reflexive practice in the fifth conception, participants not only emphasized mindful presence regarding their own manner of providing services, but also empathic responsiveness to how tenants experienced their practice. Lack of empathy is firmly established as making service users feel humiliated [56] and leading to aggression in mental health settings [55], while it is suggested that staff who empathize with service users’ feelings recognize their individuality and uniqueness promote lowered potential for conflict [65].

In their research on experiences of humiliation in mental health services, Husum et al. [56] identified that service users convey experiences of vast differences in perspectives between staff and themselves. Some practitioners are described as unwilling to explore their points of view, and are experienced as condescending and having “a top-down attitude towards them” [56 p. 151]. By adopting a dialogic stance, promoting more equality in decisions on practice, the participants voicing the sixth conception, “Involvement and dialogue” conveyed appreciation of tenants’ perspectives and involved these in drawing up preventive and management measures. By engaging with tenants, the practitioners are able to ‘look beyond’ the behaviors tenants present and commit themselves to an open investigation of the meaning behind their aggression. In this context, Gamme and Bengtsson [66] recommend professionals to integrate insights from the service user perspective with professional perspectives when devising practices aimed to mitigate the risk of violence in community mental health care. Lim et al. [63] have identified service user involvement and staff practices helping to enable service users to be active managers of their own recovery as key principles of recovery-oriented care for persons perceived at risk for aggression and violence in mental health settings. By engaging with tenants and including their perspectives in prevention and management of staff-directed aggression, a common basis for mental health practice can be realized, less inclined toward staff violating service user autonomy and fostering aggression. Being treated as equals is perceived by service users to prevent experiences of disempowerment and the subsequent use of coercion by mental health staff [67].

From an inpatient mental health setting, Carlsson et al. [61] have pinpointed ‘detached impersonal care’ as being a form of practice commonly adopted by staff faced with service user aggression. In the present study, we see this exemplified when staff preferred to observe tenants from a distance, and favored withdrawal and impersonal relations with them as a way to avoid being subjected to staff-directed aggression. This practice is largely disapproved by service users, and is seen as contributing to violence [55, 61]. Our study participants’ emphasis on proximity, equality and respect in the helping relationship as important in preventing staff-directed aggression seems to agree with principles of authentic personal care [61]. In our view, considering aggression as defiance and non-compliance to confining systems and degrading care further expresses a commitment to the service user perspective and reveals a willingness for critical reflection and self-appraisal that effectively is far removed from the internal explanations of staff-directed aggression that are more in line with descriptions of detached impersonal care.

### Conceptual change

A positive outcome of the local education and training was that four participants eventually became able to verbalize aspects of practice in line with the highest ranked conception in this study by the time of the second interview. Dialogical, involving and reciprocal conceptions of practice evident in the sixth category have earlier been found to correlate with recovery-oriented practice [68]. Being that recovery perspectives have been key in the education and training activities described in this study, we find it likely that the conceptual development experienced by the four participants moving from the fifth to the sixth category, have been supported by partaking in the municipal competence development activities.

Despite this, the instances of conceptual change we were able to identify in the present study have been moderate. We argue that the two participants voicing aspects of the first conception that they had not previously expressed did not experience a deterioration in their understanding of the phenomenon, since they maintained, and one even expanded on, their initial conceptions throughout both interviews. They appeared instead to have gained insight into what could be considered basic components of practice that also have value for the prevention and management of staff-directed aggression. As we have seen, one of these participants also developed her way of seeing from the fifth category in the first interview to the sixth category by the second interview, indicating both a development in her way of seeing and a deepening of her initial understanding.

In line with this and with particular regard to the difference in length between the first and second interviews, the impact from the education and training appeared to be mainly horizontal for most participants, rather than vertical. The education appeared to strengthen and elaborate different aspects of the phenomenon for the participants and might as such have contributed in heightening their confidence in and devotion to their particular way of seeing the phenomenon. This finding is in line with Dall’Alba and Sandberg’s [69] contention that competence and professional development oftentimes entail an elaboration and deepening of practitioners’ previously developed ways of seeing instead of a more transformative restructuring of the meaning and focus within these.

Perceived safety is viewed a prerequisite for change in witness positioning in practitioners [49]. Given the distinct emphasis participants placed on their own disempowerment in this study, it could be that perceiving themselves as powerless in the face of staff-directed aggression and service user non-compliance, and thus in an unsafe position, provided staff with few incentives to change their positions or develop elevated comprehensions of practice.

Additionally, Needham argues that ‘habituation’ is a perceptive mechanism in mental health nursing staff’s experiences of staff-directed aggression [70]. Awareness is a necessary condition for conceptual change in phenomenography [28, 38] as it is for changes in witness positioning [49]. Although several participants actually cautioned against heedlessness caused by habituation, habituation might nevertheless provide a viable explanation for how insufficient awareness in some participants might have thwarted an upwards conceptual movement following the education and training, given the relatively commonplace experience of staff-directed aggression described in our data material.

It is suggested that mental health staff experience powerful institutional pressures toward applying controlling measures toward service users [71]. Even though such practices are disputed among mental health professionals, and changes in practice are generally called for by practitioners and service users alike [51], organizational pressures and dynamics might account for some counteracting factors to conceptual and practice change, particularly regarding disempowerment-sensitive and recovery-oriented prevention and management of staff-directed aggression.

## Limitations

Even though we applied a sampling strategy judged suitable for maximizing experiential variation in this study, we concede that another sample might have generated other descriptive categories or conceptual distributions and patterns of conceptual change among participants. As we approached the participants via the supported housing managers, we cannot be sure whether they forwarded our request to select staff members, potentially biasing the findings. Yet, given the evident variation in our sample, we are confident that sampling bias have been minimal in the present study.

Another limitation is that the developed categories are based on participants’ descriptions of practice and therefore not tested empirically. However, Marton [38] argues for a strong link between peoples’ descriptions of practice and actual practice; we “act in accordance with what we see (or experience). Hence, powerful ways of acting go with powerful ways of seeing” [38, p. 83]. By this reasoning, we claim that the descriptive categories presented in this study provide a credible representation of participants’ practices, and that the higher ranked conceptions are associated with more competent prevention and management of staff-directed aggression.

An additional limitation concerns the chosen methodology for this study. The qualitative design has made it possible for us to go into detail and develop a deeper understanding of the phenomenon and the conceptual development among participants. Yet, quantitative tools could among other, have contributed to our knowledge concerning the distribution of staff-directed aggression, perpetrator and victim characteristics and the various forms of violence and aggression in municipal supported housing facilities in mental health. With regard to the aforementioned knowledge-gap regarding violence in community and non-institutional mental health settings and the ongoing deinstitutionalization trend, this would preferably be a prioritized area of research in the years to come.

Lastly, a possible limitation in this study is the empirical foundation of our discussion, being that it rests considerably on research from inpatient mental health settings, mainly because of the lack of research on staff-directed aggression in comparable supported community housing. However, due to the similarities between supported housing facilities and psychiatric wards [5], we still argue that the findings from the latter setting can help to illuminate the former, at least until a more solid empirical foundation has been established for services in supported community housing.

### Concluding remarks

This study has aimed at exploring and describing staff conceptions of practice in aggressive encounters with tenants in supported community housing, as well as inquiring into how such conceptions develop following locally based education and training. We argue that the findings of this study contribute to the evolving exploration of perspectives and experiences with staff-directed aggression in mental health services, thus supplementing’the collective mind’ [26] regarding the phenomenon. In line with qualitative research being aimed at the contextual features and complexities of a phenomenon [72], and thus being a preferable vantage point from which to establish causation [30], we also consider our findings as giving a valid indication of the effect of education and training for the participants. Overall, we found a moderate, but arguably beneficial, influence from education and training.

Our results concur with previous findings [68] in exhibiting considerable variation in understandings and experiences of prevention and management of practices among staff in municipal mental health services. In line with phenomenography, it is suggested that in order to enhance participants’ knowledge and skills, instructors must take this variation into consideration when devising education and training. In teaching sessions, it is also advisable to exploit this variation in efforts to open up aspects of the learning material for the learners [28, 38], by engaging in discussions and exchanges of perspectives and experiences with participants.

There is, however, an urgent need for knowledge on non-institutional aggression and violence toward staff in various community mental health settings. A potential way to pursue further expansion of the knowledge base would be an empirical study of various forms of conceptualization and practice with regard to outcomes of prevention and management of staff-directed aggression.

Research should, in our view also increasingly include perspectives and experiences of service users regarding practice, in order to create a comprehensive and credible foundation for knowledge-based practice in encounters with staff-directed aggression and violence in mental healthcare.

## Data Availability

The data on which this paper is based has not been made generally available, other than the parts of the data material given as illustrative quotations in the text. This is due to the need to protect the anonymity of the participants.

## References

[1] Fakhoury WKH, Murray A, Sheperd G, Priebe S (2002). Research in supported housing. Soc Psych Psych Epid..

[2] Norwegian Ministry of Health and Care Services. St.prp. 63 (1997–98). Om opptrappingsplan for psykisk helse 1999–2006. Proposition to the Storting. On an action plan for mental health. https://www.regjeringen.no/no/dokumenter/stprp-nr-63-1997-98-/id201915/. Accessed January 22 2020. **(In Norwegian)**.

[3] Hansen ILS, Øverås S. Bolig for personer med psykisk lidelse og rusproblematikk. Accommodation for persons with mental health illness and substance use problems. In: I E. Brodtkorb E, Rugkåsa M, editors. Under tak—mellom vegger. Perspektiver på boligens betydning i velferdsstaten. Under roofs—between walls. Perspectives on the value of residency in the welfare state. p. 93–111. Oslo: Gyldendal Akademisk; 2007. **(In Norwegian)**.

[4] McPherson P, Krotofil J, Killaspy H (2018). What works? toward a new classification system for mental health supported accommodation services: the Simple Taxonomy for Supported Accommodation (STAX-SA). Int J Env Res Pub He..

[5] Dyb E. Prosjekt Bostedsløse. Evaluering av et fireårig nasjonalt prosjekt. (Project Homeless. Evaluation of a four-year long national project). Oslo: Byggforsk; 2005. **(In Norwegian)**.

[6] Johnson K, Desmarais SL, Tueller SJ, Grimm KJ, Swartz MS, Van Dorn RA (2016). A longitudinal analysis of the overlap between violence and victimization among adults with mental illnesses. Psychiatry Res.

[7] Monahan J, Steadman HJ, Silver E, Appelbaum PS, Robbins PC, Mulvey EP, Grisso RLH, Banks TS (2001). Rethinking risk assessment: the MacArthur study of mental disorder and violence.

[8] Bengtsson-Tops A, Ehliasson K (2011). Victimization in individuals with psychosis: a Swedish cross-sectional study. J Psychiatr Ment Hlt..

[9] Latalova K, Kamaradova D, Prasko J (2014). Violent victimization of adult patients with severe mental illness: a systematic review. Neuropsychiatr Dis Treat..

[10] Nolan P, Dallender J, Soares J, Thomsen S, Arnetz B (1999). Violence in mental health care: the experiences of mental health nurses and psychiatrists. J Adv Nurs.

[11] Fry AJ, O’Riordan D, Turner M, Mills KL (2002). Survey of aggressive incidents experienced by community mental health staff. Int J Ment Health Nurs..

[12] Hagen IM, Svalund J. Vold, trusler og trakassering i helse- og sosialsektoren. Violence, threats and harassment in the health and social care sector. Oslo: Fafo; 2019. Report No. 32. **(In Norwegian)**.

[13] Campbell C (2017). Incident reporting by health-care workers in noninstitutional care settings. Trauma Violence Abus..

[14] Bulgari V, Ferrari C, Pagnini F, de Girolamo G, Iozzino L (2018). Aggression in mental health housing facilities: a systematic review and meta-analysis. Aggress Violent Behav..

[15] Arnetz JA, Arnetz BB (2001). Violence toward health care staff and possible effects on the quality of patient care. Soc Sci Med.

[16] Barling J, Rogers AG, Kelloway EK (2001). Behind closed doors: in-home workers’ experience of sexual harassment and workplace violence. J Occup Health Psychol.

[17] Lanctôt N, Guay S (2014). The aftermath of workplace violence among healthcare workers: a systematic literature review of the consequences. Aggress Violent Beh.

[18] Galinsky T, Feng H, Streit J, Brightwell W, Pierson K, Parsons K, Proctor C (2010). Risk factors associated with patient assaults of home healthcare workers. Rehabil Nurs..

[19] Swanson JM, Borum R, Swartz M, Hiday V (1999). Violent behavior preceding hospitalization among persons with severe mental illness. Law Hum Behav.

[20] Aberhalden C, Hahn S, Bonner YDB, Galeazzi GM, Richter D, Wittington R (2006). Users’ perceptions and views on violence and coercion in mental health. Violence in mental health settings: causes, consequences, management.

[21] Svalund J. Vold og trusler om vold i offentlig sektor (Violence and threats of violence in the public sector). Oslo: Fafo; 2009. Report No. 30. **(In Norwegian)**.

[22] Price O, Baker J (2012). Key components of de-escalation techniques: a thematic synthesis. Int J Ment Health Nurs..

[23] Lillevik OG, Øien L. Miljøterapeutisk arbeid i møte med vold og aggresjon. (Milieu-therapeutic practice in encounters with violence and aggression). Oslo: Gyldendal Akademisk; 2014. **(In Norwegian)**.

[24] Kaplan SG, Wheeler EG (1983). Survival skills for working with potentially violent clients. Soc Casework.

[25] Le Boutillier C, Leamy M, Bird VJ, Davidson L, Williams J, Slade M (2011). What does recovery mean in practice? a qualitative analysis of international recovery-oriented practice guidance. Psychiatr Serv.

[26] Marton F (1981). Phenomenography—describing conceptions of the world around us. Instr Sci.

[27] Micari M, Light G, Calkins S, Streitwieser B (2007). Assessment beyond performance: phenomenography in educational evaluation. Am J Eval..

[28] Marton F, Booth S (1997). Learning and awareness.

[29] Lum G (2013). Competence: a tale of two constructs. Educ Philos Theory..

[30] Anjum RL, Mumford S (2018). Causation in science and the methods of scientific discovery.

[31] Marton F (1986). Phenomenography: a research approach to investigating different understandings of reality. J Thought.

[32] Sandberg J, Targama A (2007). Managing understanding in organizations.

[33] Gergen KJ (2011). Relational being: Beyond self and community.

[34] Graneheim UH, Lindgren B, Lundman B (2017). Methodological challenges in qualitative content analysis: a discussion paper. Nurse Educ Today.

[35] Borg M. “Intet om oss uten oss”. (“Nothing about us, without us”). In: Borg M, Kristiansen K, editors. Medforskning—å forske sammen for kunnskap om psykisk helse (Co-operative inquiry—to inquire in collaboration for knowledge on mental health). Oslo: Universitetsforlaget; p. 29–41. 2009. **(In Norwegian)**.

[36] Sandelowski M (1995). Sample size in qualitative research. Res Nurs Health.

[37] Åkerlind GS (2012). Variation and commonality in phenomenographic research methods. High Educ Res Dev..

[38] Marton F (2015). Necessary conditions of learning.

[39] Sjöström B, Dahlgren LO (2002). Applying phenomenography in nursing research. J Adv Nurs.

[40] Guba EG, Lincoln YS (1989). Fourth generation evaluation.

[41] Rosetto KR (2014). Qualitative research interviews: assessing the therapeutic value and challenges. J Soc Pers Relat..

[42] Bowers L, Nijman H, Allan T, Simpson A, Warren J, Turner L (2006). Prevention and management of aggression training and violent incidents on U.K. acute psychiatric wards. Psychiat Serv..

[43] Duxbury J (2002). An evaluation of staff and patient views of and strategies employed to manage inpatient aggression and violence on one mental health unit: a pluralistic design. J Psychiatr Ment Hlt..

[44] Paterson B, Leadbetter D, Miller G, Bowie V (2010). Re-framing workplace violence directed towards nurses in mental health services in the UK: a work in progress. Int J Soc Psychiatr..

[45] Njiman HLI, Joost MLG, àCampo MD, Ravelli DP, Merckelbach HLGJ (1999). A tentative model of aggression on inpatient psychiatric wards. Psychiatr Serv..

[46] Whittington R, Richter D, Richter D, Wittington R (2006). From the individual to the interpersonal: environment and interaction in the escalation of violence in mental health settings. Violence in mental health settings: causes, consequences, management.

[47] International Council of Nurses, Public Services International, World Health Organization and International Labour Office. Framework for guidelines for addressing workplace violence in the healthcare sector—the training manual. Geneva, World Health Organization. https://www.ilo.org/wcmsp5/groups/public/—ed_protect/—protrav/—safework/documents/instructionalmaterial/wcms_108542.pdf. Accessed January 23 2020.

[48] Paterson B, Leadbetter D, Miller G (2005). Beyond Zero Tolerance: a varied approach to workplace violence. Br J Nurs..

[49] Weingarten K (2003). Common shock Witnessing violence everyday: how we are harmed, how we can heal.

[50] Björkdahl A, Palmstierna T, Hansebo G (2010). The bulldozer and the ballet dancer: aspects of nurses’ caring approaches in acute psychiatric intensive care. J Psychiatr Ment Hlt..

[51] Duxbury J, Whittington R (2005). Causes and management of patient aggression and violence: staff and patient perspectives. J Adv Nurs.

[52] Bowie V, Privitera M (2010). An emerging awareness of the role organizational culture and management style can play in triggering violence. Workplace violence in mental health and general healthcare settings.

[53] Shaw MMC (1998). Nursing home resident abuse by staff: exploring the dynamics. J Elder Abuse Negl..

[54] Shaw MMC (2004). Aggression toward staff by nursing home residents: findings from a grounded theory study. J Gerontol Nurs..

[55] Gudde GB, Olsø TM, Whittington R, Vatne S (2015). Service users’ experiences and views of aggressive situations in mental health care: a systematic review and thematic synthesis of qualitative studies. J Multidiscipl Healthc..

[56] Husum TL, Legernes E, Pedersen R (2019). A plea for recognition: users’ experiences of humiliation during mental health care. Int J Law Psychiat..

[57] Hartling LM, Lindner E, Spalthof U, Britton M (2013). Humiliation: a nuclear bomb of emotions?. Psicol Polít.

[58] Vatne S, Fagermoen MS (2007). To correct and to acknowledge: two simultaneous and conflicting perspectives of limit-setting in mental health nursing. J Psychiatr Ment Health Nurs.

[59] Bowers L. A model of de-escalation Ment Health Pract. 2014;17(9):36–7. doi:10.7748/mhp.17.9.36.e924.

[60] Duperouzel H (2008). ‘It’s ok for people to feel angry’: the exemplary management of imminent aggression. J Intell Disabil..

[61] Carlsson G, Dahlberg K, Ekebergh M, Dahlberg H (2006). Patients longing for authentic personal care: a phenomenological study of violent encounters in psychiatric settings. Iss Ment Health Nurs..

[62] Price O, Baker J, Bee P, Grundy A, Scott A, Butler D, Cree L, Lovell K (2017). Patient perspectives on barriers and enablers to the use and effectiveness of de-escalation techniques for the management of violence and aggression in mental health settings. J Adv Nurs.

[63] Lim E, Wynaden D, Heslop K (2019). Changing practice using recovery-focused care in acute mental health settings to reduce aggression: a qualitative study. Int J Ment Health Nurs..

[64] Lillevik OG, Øien L. Kvaliteter hos hjelperen som bidrar til å forebygge trusler og vold fra klienter. Qualities of the helper contributing to prevent threats and violence from clients. Nord Tidsskr Helseforsk. 2010;6(2):84–96. 10.7557/14.1191. **(In Norwegian)**.

[65] Lim E, Wynaden D, Heslop K (2019). Consumers’ perceptions of nurses using recovery-focused care to reduce aggression in all acute mental health including forensic mental health services: a qualitative study. J Recov Ment Health..

[66] Gamme M, Eriksson BG (2018). Promoting personal growth and balancing risk of violence in community-based mental health care: a professional perspective. Sage Open..

[67] Einbu M, Larsen IB (2016). Innenfrakunnskap om årsaker til truende atferd eller aggresjon og alternativer til bruk av tvangsmidler. Insider knowledge on causes to threatening behaviors or aggression, and alternatives to coercive measures. Tidsskr Psyk Helsearb..

[68] Maagerø-Bangstad ER, Sælør KT, Ness O (2019). Encountering staff-directed aggression in mental health and substance abuse services: exploring conceptions of practice following education. Int J Ment Health Sy..

[69] Dall’Alba G, Sandberg J (1996). Educating for competence in professional practice. Instr Sci..

[70] Needham I, Richter D, Wittington R (2006). Psychological responses following exposure to violence. Violence in mental health settings: causes, consequences, management.

[71] Gudjonson GH, Rabe-Hesketh S, Szmukler G (2004). Management of psychiatric in-patient violence: patient ethnicity and use of medication, restraint and seclusion. Brit J Psychiat..

[72] Creswell JW, Poth CN (2018). Qualitative inquiry & research design: choosing among five approaches.

